# Hopamine as Personalized Medicine for Persons with Parkinson’s Disease

**DOI:** 10.3233/JPD-230012

**Published:** 2023-03-14

**Authors:** Marina A. Noordegraaf, Sanne W. van den Berg, Bastiaan R. Bloem

**Affiliations:** a Patient Advocate and Patient Researcher at the Dutch Parkinson’s Association, The Netherlands; b Radboud University Medical Centre; Donders Institute for Brain, Cognition and Behaviour; Department of Neurology; Centre of Expertise for Parkinson & Movement Disorders, Nijmegen, The Netherlands

**Keywords:** Dopamine, hopamine, hope, Parkinson’s disease, personalized care management, personalized medicine

## Abstract

Prescribing dopamine replacement therapy remains the most common approach used by physicians who strive to support persons with Parkinson’s disease. In this viewpoint, we argue that instead of merely prescribing dopamine, healthcare professionals should particularly encourage and enable persons with Parkinson’s disease to draft their own personalized prescription of “*hopamine*”. The term hopamine is a self-invented neologism representing the uniquely personal set of hopes, desires, experiences, and skills of each individual with a dopamine deficit. As such, the concept of hopamine–as a reflection of the unique personal characteristics of each person with Parkinson’s disease—really supplements that of dopamine–as a reflection of each person’s unique physical characteristics. Whereas a prescription of dopamine replacement medication necessitates the diagnosed individual to lay his or her fate in the hands of medical professionals, adding a personalized dose of hopamine to the therapeutic mix empowers persons to self-manage daily life with Parkinson’s disease. In this viewpoint, we argue that hopamine is a prerequisite for personalized medicine and offer several practical recommendations for how medical professionals can introduce the concept of hopamine in daily clinical practice.

“*Some modicum of hope is necessary for carrying on with daily living. At the same time, hope represents a daunting source of vulnerability*.” *[1]*

## THE DYNAMICS OF HOPE

A significant proportion of persons with neurodegenerative conditions such as Parkinson’s disease are dissatisfied with the delivery of their diagnosis [2, 3]. The newly diagnosed individual typically feels disempowered and expresses a clear need to regain control [4]. More often than not, people diagnosed with Parkinson’s disease have no idea what the label they have just been given is all about. The five devastating words “You have got Parkinson’s disease” are typically delivered during relatively brief consultations, without sufficient time to address emotions or to share relevant information, leaving the affected individual and their loved ones behind in bewilderment and despair. A moment when all hope seems to have been lost. Being in such a dependent and literally hopeless position makes it much easier, if not inevitable, to be drawn into hope dynamics where the physician knows best; depending on the healthcare setting, this is sometimes the family practitioner, but more often a medical specialist, such as a neurologist or—in countries such as the UK—a geriatrician.

What physicians know all too well when they diagnose someone with Parkinson’s disease is that this person has just been given a life-long sentence. Currently, there is no turning back, no cure, no quick fix. But at the same time, physicians should also realize that for increasingly many individuals with Parkinson’s disease, basing their hope on dopamine replacement therapy, which has been the cornerstone of the treatment of Parkinson’s disease since the late 1960 s, will simply not be enough. John Roche, a man who lives with Parkinson’s, tweeted on World Parkinson’s Day 2021: “If I walked into a car showroom or kitchen outlet and they offered me the same goods that were available forty years ago, I would turn around and walk out. Why do we have to put up with this in respect of Parkinson’s medication - come on, something is not right there.”

Some physicians may not want to give any false hope and tell the freshly diagnosed that the most realistic scenario is that of an initial ‘honeymoon’ (a relatively good period lasting for about 5 to 10 years), after which the symptoms and corresponding disability will undoubtedly worsen quickly. Such a message, paradoxical and dissociating in itself, is based on group level data from the past. In fact, the very use of the word ‘honeymoon’ is definitely not one that will resonate with many persons who have just been dealt such a bad card. Hope, however, is a personalized commodity with value in the future only. More future-oriented physicians may want to soften the blow and attempt to install hope beyond what is currently known, for example by pointing to outliers with a more benign prognosis, or by pointing to the tangible progress that is being made in the scientific field, emphasizing that the insights into the underlying neurodegenerative processes are growing fast, that the genetic contribution to the various forms of Parkinson’s disease is increasingly being understood, that the first interventions with disease-modifying potential are being explored (regular exercise being perhaps closest to delivering the promise of slowing disease progression, and also one that can be applied to virtually everyone, regardless of the form of Parkinson’s disease each individual may have), that neurosurgical interventions continue to be improved, that stem cell therapies are being refined, and that integrated person-centered models of care are currently taken to the test in living field labs. They may even share the words of the late and great Tom Isaacs, founder of Cure Parkinson’s and an acknowledged optimist, who felt that, because of these advances in neurosciences, this is a time of cautious hope. In doing so, these physicians attempt to create new hopes that may once take shape in a galaxy far away. However, that galaxy that does not align with the one to which the freshly diagnosed will be returning to seconds after they leave the room where the diagnosis was delivered. And even though the latter approach certainly seems to be the more empathetic way of delivering a diagnosis, this type of hope narrative may backfire when the subsequent progression of symptoms does not keep pace with the physician’s hopes.

These physicians obviously mean well. They hope to live to see the day when the relentless disease progression is stopped, reversed or when better symptomatic support is available for each affected person in the world. But this biomedical narrative of hope [5] inevitably is and remains the perspective of the doctor. Sooner or later, people with Parkinson’s disease will need to develop their own personalized hope narratives, with ingredients they can feed themselves on a daily basis. For the beauty of hope is that it simply cannot be delegated. Not without losing its form and strength.

## PREVENTING THE DELEGATE HOPE TRAP

Hope is a substantiated expectation that one can influence one’s own future in a positive way [6, 7]. However, when hope is delegated, any actionable ‘hope potential’ invariably transforms into a more passive form. A person with Parkinson’s can decide to trust or distrust the physician’s judgment, to be optimistic, pessimistic, or anything in between. But the actionable component that is inherent to hope leaves the premises right at the moment when a physician prescribes any type of dopaminergic medication. Hope is now externalized, leaving the person with Parkinson’s in a passive state, awaiting the forthcoming and hopefully positive effects of the prescribed medication. In the words of a person diagnosed with amyotrophic lateral sclerosis: “Once you put the responsibility for your fate in the hands of an outside force, you are out of the game” [8].

Not considering the personal hope of someone with Parkinson’s disease in the consultation room comes at a high cost, considering that feelings of hopelessness are more strongly correlated to quality of life than physical functioning [9]. Despite all of their good intentions, physicians are vulnerable to falling for the ‘delegate hope trap’ (a term based on the ‘Never delegate understanding‘ podcast series). But when the trap opens, they find themselves in an unsustainable position, especially when considering that they may only see their patients for several hours a year [10].

## HOPAMINE AS PERSONALIZED MEDICINE

**Fig. 1:**
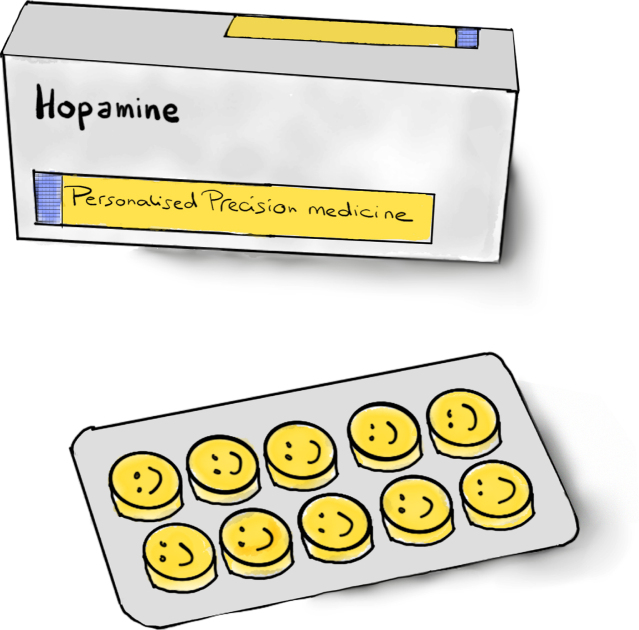
A pillbox containing hopamine, to symbolize the crucial need to address the issue of hope on a personalized basis for each individual living with Parkinson’s disease, starting immediately from the diagnosis onwards and redrafting this during the constantly changing course of Parkinson’s disease (drawing by Marina Noordegraaf).

Here, we argue that healthcare professionals should actively encourage people diagnosed with Parkinson’s disease to talk about their personal hope and enable them to draft their own personalized prescription of *hopamine* (Fig. 1). The term hopamine is a self-invented neologism (“*hope of mine*”) and represents the uniquely personal set of hopes, desires, experiences, and skills of each individual with a dopamine deficit. As such, the concept of hopamine—as a reflection of the unique *personal* characteristics of each person with Parkinson’s disease—really supplements that of dopamine, as a reflection of each person’s unique *physical* characteristics. Personalized medicine can only be established when dopamine and hopamine are considered equally. Specifically, this means that two individuals might present with the same clinical presentation of Parkinson’s disease, in terms of, e.g., age, gender, and symptoms, yet they may make very different choices with respect to pharmacotherapy or other management options as a result of their uniquely personal hopes, wishes, and abilities. So, the personalized dose of hopamine may apply to predominantly motor features for some, but predominantly to non-motor features for others. Therefore, health care professionals need to look through both the dopamine and the hopamine lens to see the person with Parkinson’s as a whole and formulate a personalized care plan.

The concept of hopamine thus fits well within a broader shift that is taking place within healthcare, namely that from illness to wellness, including the transition from asking ‘What is the matter with you?’ to ‘What matters to you?’ [11]. Instead of merely prescribing dopamine replacement medication that lays the fate of the diagnosed individual in the hands of medical professionals, actively inviting and supporting each individual to formulate their own personalized dose of hopamine might help to avoid the risk of creating false hopes and a sense of hopelessness. As such, hopamine might offer a new tool and viewpoint to support the complex adjustment process that helps individuals with Parkinson’s disease to maintain a stable sense of self, feel in control and hold a positive mindset, despite illness changes and ongoing deterioration [12].

## RECOMMENDATIONS FOR CLINICAL PRACTICE TO GROW, FOSTER, AND SPREAD HOPAMINE

The good news is that physicians can consciously decide to step aside. Being the first point of entry into unknown territory for any newly diagnosed individual, physicians are uniquely positioned to give persons with Parkinson’s disease a big head start in unlocking resources of hope. But conveying the crucial message of hopamine must not be restricted to just the physician who is prescribing dopaminergic medication, but should really be embraced by any healthcare professional who is involved in the care for families with Parkinson’s disease. Indeed, discussing personal hopes, wishes, and skills in the light of future perspectives are perhaps even better addressed by, e.g., Parkinson nurses or social workers, but could equally be discussed by an allied health therapist who throughout the treatment period typically builds up an intimate and powerful relation with an individual with Parkinson’s disease.

To address and foster hopamine, healthcare professionals may combine the following intertwined approaches.

### Facilitating disease literacy and education

It is extremely difficult to draft a personalized prescription of hopamine if you do not know what you are up against [13–16]. Considering that low health literacy in Parkinson’s disease is associated with adverse outcomes [17], physicians (and any other healthcare professionals) should not shy away from painting a picture of Parkinson’s disease that is as complete as possible, and point to trusted information and possible self-empowerment courses for further discovery. For example, national Parkinson associations or funding bodies like the Michael J. Fox Foundation are examples of rich sources of relevant information about the nature of Parkinson’s disease and its management. The ‘first steps’ program of Parkinson’s UK is a great example of an online program designed to get the recently diagnosed back in the saddle as soon as possible after the diagnosis has been conveyed [18]. In addition to explaining the nature of Parkinson’s disease, healthcare professionals also should proactively educate individuals about the importance of hopamine as a prerequisite to personalize individual care plans.

### Pointing to options for self-management

Giving an overview of evidence-based avenues for self-management allows persons with Parkinson’s disease to select the ones that resonate with their personalized hopamine recipe. Examples include engaging in regular physical activities, participating in stress-alleviating interventions like mindfulness or yoga [19], taking up dance, changing dietary habits, moving ‘big’, seeking psychological treatment (such as a positive psychology approach) [20], looking for peer support, identifying peer-led education [18], and many others.

A part of self-management is the recent move towards self-assessment, for example using diaries or wearable sensors, as a basis for personalized disease management [10]. Being able to track your own disease can be a way of regaining control, although it may come with a price, because self-tracking can be time-consuming and imply that individuals are confronted continuously with their own disease [10]. The choice to resort to self-tracking as a way to self-manage is therefore clearly a personalized one.

### Sitting back and tuning in

Upon the diagnosis, physicians have no other choice than to bring life-changing and disempowering news. But they do have the choice to make the ensuing parts of the consultation about seeing the person behind the diagnosis, before deciding on personalized treatment options. Each individual living with Parkinson’s disease not only has his or her unique type of Parkinson’s disease (dopamine) [21], they also have unique lives, hopes, desires, experiences, and skills (hopamine). Understanding that a diagnosis happens right in the middle of a life, with a story of its own, may help uncover unique talents, strengths, and resources. It is exactly because the outcome of everyone’s Parkinson’s journey is uncertain that people will need a personalized recipe of hopamine to self-manage their life and to deal with an uncertain future with Parkinson’s disease. For medical professionals, learning to be at ease with such uncertainty is essential to let hope grow [5]. Sitting back, tuning in, and fostering curiosity is needed in the communication styles of health care professionals to understand personalized guidance which different persons may need to access their individual resources and draft their individual hopamine recipe.

## CONCLUSION

Investigations into hope-enhancement as a therapeutic target are promising [20, 22]. There is even a research unit, Hope Studies Central, that is explicitly devoted to the study of hope in human living, and their work includes a section on Parkinson’s disease [23]. The significant beneficial effects that patients experience from self-management strategies speaks volumes [24]. By stressing the importance of hopamine from the diagnosis onwards, medical professionals send a powerful signal that they see the newly diagnosed as autonomous, as someone who is able to adjust and integrate Parkinson’s disease into their life in a very personalized way.

Enabling hopamine along with, or better even *before* dopamine, might just be the essential step to overcoming the disempowering experience of a diagnosis of Parkinson’s disease as early as possible and thereby improve the affected individual’s quality of life accordingly.

Importantly, hopamine is the penultimate form of personalized medicine, as it represents each individual’s uniquely personal hopes, preferences, and abilities (see the examples in Panel 1). Personal hope is dynamic, individual, and context-driven [1], and so does hopamine change with the tides of disease progression and life events. Adding hopamine to the therapeutic mix empowers persons to self-manage daily life with Parkinson’s disease. So, in addition to their mere daily intake of dopamine replacement therapy, we wish the millions of persons with Parkinson’s disease in the world will take their own personalized daily dose of hopamine.

We hope that this viewpoint will encourage medical professionals and persons with Parkinson’s disease worldwide to co-create the fertile soil where hope can grow and where we can inspire each other to formulate our personalized dose recipe of hopamine as sharp as possible.

## Appendix

**Panel 1. Examples of personalized hope (hopamine).** Each of these individual’s own recipes for hopamine is depicted as follows: If I do [A] –which I desire because of [B] and which lies within my reach because of [C] –I hope it will have effect [D] on the Parkinson’s of [E]. The individual recipes for hopamine will be different for each individual, and these may change with live events or disease progression. For example, a newly diagnosed individual may be paralyzed by fear and the sole personal hope might be that the physician is wrong about the diagnosis. For another person with Parkinson’s disease, personal hopamine constitutes the wish to continue baby-sitting the grandchildren without too much hindrance for as long as possible. Note that the examples of collective hopamine reflect the hopes of individuals who have learned about the diagnosis years earlier, and who have adjusted to life with Parkinson’s.

Examples of individual hopamine recipes

**Marina Noordegraaf: I hope to see my children grow up**

*If I cycle on my spinning bike for half an hour every day* –*which I desire because there is evidence it might positively influence the course of my Parkinson*’*s, and which is within my reach because the spinning bike was 30 dollars only and is patiently waiting for me at my bedside every morning when I wake up* –*I hope it will slow down my Parkinson*’*s progression and enable me to see my children grow up.*

**(Imaginary) carer of a person with Parkinson’s: I hope to support my partner with Parkinson’s without losing myself**

*If I retire early* –*which I desire because I am very tired of combining the care of my partner with Parkinson*’*s and my full-time job, and which lies within my reach because we have the finances covered* –*I hope to make the necessary space in myself to be able to support my partner with Parkinson*’*s and contribute to increasing our joint quality of life.*

Examples of collective hopamine recipes:

**Bas Bloem: I hope to see the day when a cure is born**

*If I share what gives me hope according to the latest biomedical developments* –*which I desire because I want to prevent unnecessary disability in my patients, and which lies within my reach because of my education and up-to-date knowledge (disease literacy)* –*I hope this allows persons with Parkinson*’*s to see what I see: intertwined paths of unmistakable progress that give me hope that tangible benefits will arrive for people with Parkinson*’*s in a not-too-distant future.*

**Marina Noordegraaf: I hope to help prevent unnecessary cases of Parkinson’s disease**

*If I engage in advocacy on showing how pesticides may contribute to Parkinson*’*s* –*which I desire because I want to do everything in my power to prevent other persons to unnecessarily get Parkinson*’*s, and which lies within my reach because I have a background in organic chemistry combined with a systemic view on world problems* –*I hope this will help activate our collective awareness towards more sustainable agricultural practices where a reduced dependency on pesticides and less cases of Parkinson*’*s go hand in hand.*

**Sara Riggare: I hope to contribute to increased agency and autonomy among persons with Parkinson’s**

*If I engage in and share my endeavors in self-tracking and personal science* –*which I desire because I am the kind of person that wants to know as much as she can about my disease and treatment, and which lies within my reach because I am an engineer by education* –*I do not only help myself but I also hope to contribute to increased agency and autonomy for all persons with Parkinson*’*s, e.g. by making active involvement as an equal partner in digital health development become the new norm.*

**Tom Isaacs: I hope to become the first person to insert the words “I used to have Parkinson’s disease”**

*Hope ignites a fire in the soul and rekindles the dying embers of ambition and purpose. When people with Parkinson*’*s accept there is the chance, no matter how small, that we are not doomed to a life borne out of our worst nightmares, then we start to gather information about our Parkinson*’*s and we start to concentrate on the things we can do rather than those we cannot.*
